# A Decade of Transforming Journey with IJPS

**DOI:** 10.1055/s-0044-1801326

**Published:** 2024-12-30

**Authors:** Dinesh Kadam

**Affiliations:** 1 Department of Plastic and Reconstructive Surgery, A. J. Institute of Medical Sciences and Research Centre, Mangalore, Karnataka, India

Dear Esteemed Member,

I am pleased to present yet another print issue of the *Indian Journal of Plastic Surgery* ( *IJPS* ), in addition to the six regular issues that have already been published this year. Publishing articles in the print issue increases visibility on PubMed and enhances authors' academic credentials. We have a long lineup of *e-First* published articles awaiting publication in print issues, and this problem of “plenty” is a pleasant one that exemplifies the journal's vibrant growth and robust submissions.

With great humility and gratitude, I express that I am completing my tenure as the Editor. This has been an extraordinary journey, marked by challenges, learning, and immense growth—not only for me but also for the journal we have all nurtured together. Completing over 7 fulfilling years in this position and many more as Deputy Editor, I am filled with gratitude, pride, and a sense of accomplishment. I take this opportunity to reflect on my decade-long association with this esteemed journal, a journey filled with purpose and achievements. Together, we have witnessed several transformative milestones for the *IJPS* .

## Key Accomplishments

### Rebranding with a New Publisher

During my tenure as Deputy Editor under Prof. Mukund Jagannathan, it became evident that rebranding the journal was imperative for its global outreach. Once I assumed office in 2018, after careful negotiations, we partnered with Thieme Publishers, an internationally acclaimed name in medical publishing. Under their aegis, the *IJPS* underwent a significant transformation. The inaugural print issue was released on National Plastic Surgery Day in July 2019 at AIIMS, Delhi. The new design, with a vibrant front page and all-colored interior pages, online-first articles marked a new era for the journal. 1 This move not only elevated the journal's quality but also brought *IJPS* into the ranks of internationally recognized plastic surgery journals.

### Faster Publication: The Era of e-First Articles

The introduction of e-First publication in 2019 significantly reduced the time between article acceptance and online publication, now averaging just 10 days. 2 This has enabled authors to gain faster visibility and prompt recognition of their work.

### Doubling Yearly Issues

**Fig. 1:**
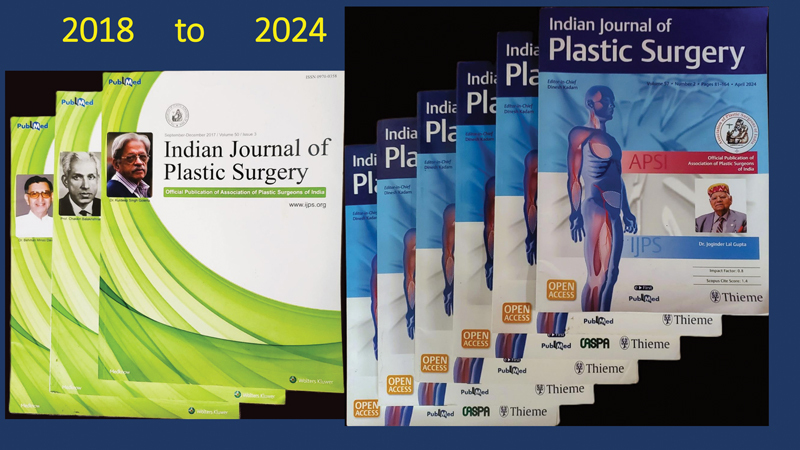
During the period from 2018 to 2024, the *IJPS* increased from three to six issues annually.

The *IJPS* began publishing twice a year in 1968, increased to three times a year in 2008, then to four times a year from 2020 to 2022, and finally to six times a year in 2023. 3 This rapid expansion from three to six a year has enabled us to publish more articles and remain competitive with international journals ( Fig. 1 ). This milestone also reflects the increased quality and quantity of submissions. Despite increased costs, we successfully managed to doubled the output, accommodating a higher volume of quality articles and keeping pace with leading international titles.

### Achieving the First Impact Factor

The impact factor (IF) is the most important journal metric and a goal for every journal editor. A historic milestone was reached in 2022 when the *IJPS* received its first-ever IF after 56 years of publication. 4 This achievement underscores the journal's credibility and growing influence in the global academic community.

### Publishing Archival Content

**Fig. 2:**
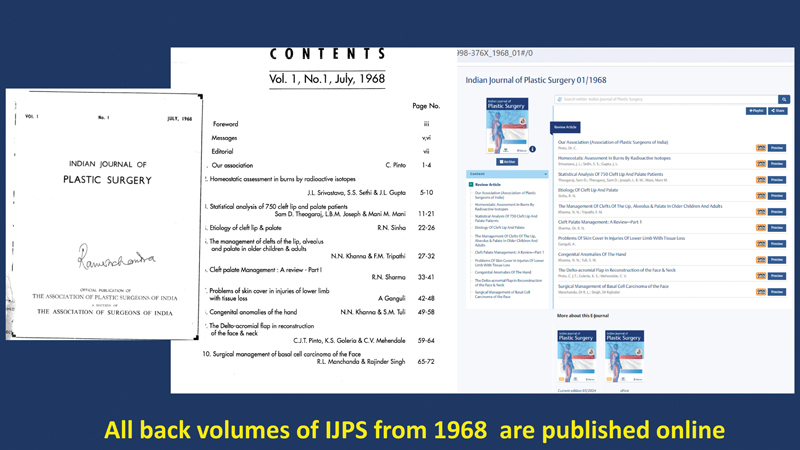
The table of contents of the inaugural print issue of the *IJPS* in 1968 converted to digital format and republished with a fresh doi.

This is the most fulfilling accomplishment, dear to my heart. It was a defining achievement to digitize and publish all issues of the *IJPS* since its inception in 1968. 5 This endeavor made accessible over 30 years of invaluable articles previously lost in print-only formats ( Fig. 2 ). Now available for citation with DOI numbers, these archival issues serve as a treasure trove of knowledge and a chronicle of the Association of Plastic Surgeons of India (APSI). This could potentially redefine the history of plastic surgery with Indian contributions.

## Financial Sustainability

Despite increasing costs, we maintained financial health by generating income through subscriptions and advertisements while managing within allocated grants. The Journal Corpus' annual grant covers only 60% of the cost, leaving the rest for us to generate. Nevertheless, I am glad to mention the corpus has been doubled in the past 7 years, which will support the journal in the long term. Importantly, we remain a Diamond Open Access journal without charges to authors and readers, the only such model publication in plastic surgery with IF. 6 Further, we continued delivering print issues to members at no extra cost, preserving the tangible connection that print brings among members.

### Retaining Print Copies

Despite the odds against print copies in terms of the cost, time, and effort, we firmly believe that the benefits outweigh the losses. To me, it is the only official scientific communication that brings together the association, enables us to know each other's work, acknowledges the departments and expertise in the country, and serves as an inspiration for publication. Considering its vital role in promoting publication culture and positively impacting the members, we made concerted efforts to generate income to cover expenses. Even during challenging times, such as the COVID-19 pandemic, we ensured print issues reached members without incurring additional costs. 7 Our good intentions and actions are no match for the various limitations beyond our control, making it practically impossible to deliver to everyone every time. Nevertheless, this is an ongoing process; we continue working together to ensure its effective delivery and optimal utilization of resources. Your kind understanding and cooperation in addressing any shortcomings is greatly appreciated. I am grateful to Dr. Maneesh for assisting me with this task and keeping the delivery cost minimal.

## Innovations in Editorial Practices

*Resident editorial board:* For the first time, postgraduate residents were integrated into the editorial process, nurturing a pool of future editorial leaders.
*Diverse editorial team:* An inclusive team of brilliant associate editors from across India, along with the introduction of a Business Editor, ensured effective management and progress of the journal.
*Social media outreach:* Our presence on platforms like Facebook, Instagram, and X has grown, engaging a wider audience and increasing visibility.
*Reviewer recognition:* The introduction of reviewer awards acknowledged the critical role of our dedicated reviewers, whose efforts are often unseen but integral to the journal's success. 8

## APSI-Focused Editorials

Throughout my tenure, I have authored editorials that address the core interests of our members of APSI and trainees. These writings have highlighted the remarkable achievements of our members, thus enhancing India's reputation on global platforms. Editorial topics have included critical reflection on the Ayushman Bharat scheme, promoting World Plastic Surgery Day, India's advancements in hand transplants, and progress in aesthetic surgery. 9 10 11 12 I have also spotlighted the growth and achievements of the *IJPS* , the publication of archives, and contemporary issues such as APSI awards, membership, and research priorities. 13 14 15 16 17 These editorials, I trust, have been timely and relevant, serving as a valuable resource for our readers. 18 19 20 21 22 23 24

## Thematic Issues

We introduced dedicated theme issues, enriching the journal's content with focused topics such as lower extremity reconstruction, hair restoration, gender incongruence, aesthetic surgery, and normative data. 25 26 This has enhanced the journal's offerings, attracting diverse expertise and readership.

## Priorities to APSI Members

As the official publication of APSI, the journal has always prioritized our members' interests and contributions. We strived to support our authors by providing constructive feedback, guiding them in refining their submissions, and improve their articles. Our goal was to nurture and showcase the academic potential of our association while maintaining the journal's high standards. The review process remained rigorous yet considerate, ensuring fairness and quality. Submissions from APSI members received deserving attention, and every effort was made to facilitate their publication. However, articles that were not accepted were carefully evaluated based on thorough reviewer feedback and editorial judgment. My own diverse clinical expertise allowed me to assess each article independently and impartially. The contributions of our dedicated reviewers have been invaluable in maintaining and enhancing the journal's quality, and I remain deeply grateful to them. We take pride in the significant number of APSI members who have contributed to the *IJPS* and showcased their academic excellence.

## A Gratifying Journey and the Team Behind the Success

No journey is solitary, and this one has been no exception. Being entrusted with the responsibility of leading the *IJPS* has been intellectually enriching and personally challenging. Balancing deadlines alongside a demanding microsurgery practice and personal commitments was not easy, but it was a privilege to contribute to the journal's growth. Working with brilliant minds, esteemed institutions, and dedicated colleagues has been a rare and rewarding experience. My associate editors' dedication and hard work, the reviewers' diligence, and the authors' cooperation have been the lifeblood of this journal. I am deeply thankful to each of you for your unwavering support and contributions and for every member joining me in nurturing our journal. 27

Furthermore, the team at Thieme Medical Publishers, India, has been very accommodating, supportive, and efficient in publishing each issue on time. I remain indebted to them.

## Looking Ahead

I had the privilege of inheriting our beloved *IJPS* in its golden jubilee year, 2018, a legacy nurtured by visionary predecessors since the previous century. Over the past 7 years, the journal has evolved significantly—embracing newer publication models, rebranding with a fresh and vibrant look, publishing more frequently with a globally reputed publisher, and achieving its highest quality metric: the IF. Additionally, one of the most significant milestones was adding all previous archives, creating a robust repository of knowledge that now aligns the *IJPS* with leading international journals.

The *IJPS* ' open-access model, which ensures free publication for authors, sets it apart and positions it for remarkable growth in the future. With the enthusiastic involvement of younger members, dedicated authors, and committed reviewers, the journal is well equipped to scale new heights. I am profoundly grateful to the association and its members for entrusting me with this responsibility and allowing me to be a part of this incredible journey.

As I hand over the baton, I do so with immense pride and confidence in the future of the *IJPS* . The incoming editorial team inherits a thriving journal, and I am confident they will steer it to even greater heights. With an enthusiastic and capable editorial team led by Prof. Maneesh Singhal, I am certain the journal will continue to thrive, setting new benchmarks of excellence.

As I bid adieu, I remain deeply connected to the *IJPS* and the APSI. May the journal continue to grow, inspire, and serve as a beacon of knowledge in plastic surgery.

Long Live the *IJPS* . Long Live the APSI. Jai Hind!
